# Steroid sulfatase and sulfotransferases in the estrogen and androgen action of gynecological cancers: current status and perspectives

**DOI:** 10.1042/EBC20230096

**Published:** 2024-12-04

**Authors:** Tea Lanišnik Rižner, Marija Gjorgoska

**Affiliations:** Institute of Biochemistry and Molecular Genetics, Faculty of Medicine, University of Ljubljana, Ljubljana, Slovenia

**Keywords:** androgens, estrogens, intracrinology, sulfatase, sulfotransferase

## Abstract

Sulfatase (STS) and sulfotransferases (SULT) have important role in the biosynthesis and action of steroid hormones. STS catalyzes the hydrolysis of estrone-sulfate (E1-S) and dehydroepiandrosterone-sulfate (DHEA-S), while sulfotransferases catalyze the reverse reaction and require 3-phosphoadenosine-5-phosphosulfate as a sulfate donor. These enzymes control the concentration of active estrogens and androgens in peripheral tissues. Aberant expression of *STS* and *SULT* genes has been found in both, benign hormone-dependent diseases and hormone-dependent cancers. The aim of this review is to present the current knowledge on the role of STS and SULT in gynecological cancers, endometrial (EC) and ovarian cancer (OC). EC is the most common and OC the most lethal gynecological cancer. These cancers primarily affect postmenopausal women and therefore rely on the local production of steroid hormones from inactive precursors, either DHEA-S or E1-S. Following cellular uptake by organic anion transporting polypeptides (OATP) or organic anion transporters (OAT), STS and SULT regulate the formation of active estrogens and androgens, thus disturbed balance between STS and SULT can contribute to the onset and progression of cancer. The importance of these enzymes in peripheral estrogen biosynthesis has long been recognized, and this review provides new data on the important role of STS and SULT in the formation and action of androgens, their regulation and inhibition, and their potential as prognostic biomarkers.

## Introduction

This mini-review aims to present the current state of knowledge on the role of sulfatase (STS) and sulfotransferases (SULT) in gynecological cancers, endometrial cancer (EC) and ovarian cancer (OC).

OC and EC account for 10% of all cancers in Europe and 9% of cancer-related deaths per year [1]. EC is the most common gynecological cancer in industrialized countries [1] and OC is the most lethal gynecological malignancy [2]. These are hormone-dependent cancers, as steroid hormones, particularly estrogens, play a role in their development and progression. Androgens also have an important role, but are still less studied [3–5]. This is an update of the review published in 2016 [6] and therefore concentrates mainly on studies that have been conducted since then. It focuses on the role of STS and SULT in peripheral estrogen and androgen biosynthesis and action, their regulation and inhibition, the impact on survival of EC and OC patients and includes analysis of The Cancer Genome Atlas (TCGA) transcriptomics data.

## Sulfatase and sulfotransferase in intracine action of steroid hormones

STS and SULT regulate the biosynthesis of steroid hormones and thus play a crucial role in human homeostasis [6]. In higher primates and in humans, steroid hormones are formed in endocrine glands and also in peripheral tissues from inactive precursors of adrenal origin or *de novo* from cholesterol. Steroid hormones act in the target tissues or in the same (intracrine action) or neighboring (paracrine action) cell in which they are formed [7]. They activate corresponding intracellular receptors that regulate the expression of target genes or membrane-bound receptors, either covalently modified palmitoylated classical receptors [8] or G protein-coupled receptors (GPCR) [9] to activate intracellular signaling pathways. Steroid hormones are excreted mainly via the urine (more than 80%), but also via the bile after sulfation or irreversible glucuronidation by UDP glucuronosyl transferases in the liver [10]. In contrast with sulfates, which also serve as precursors of steroid hormones, glucuronides are the final and the main excretion products, which can only be hydrolysed by the intestinal microbiota, allowing the reabsorption of free steroid hormones [10,11].

**Figure 1:**
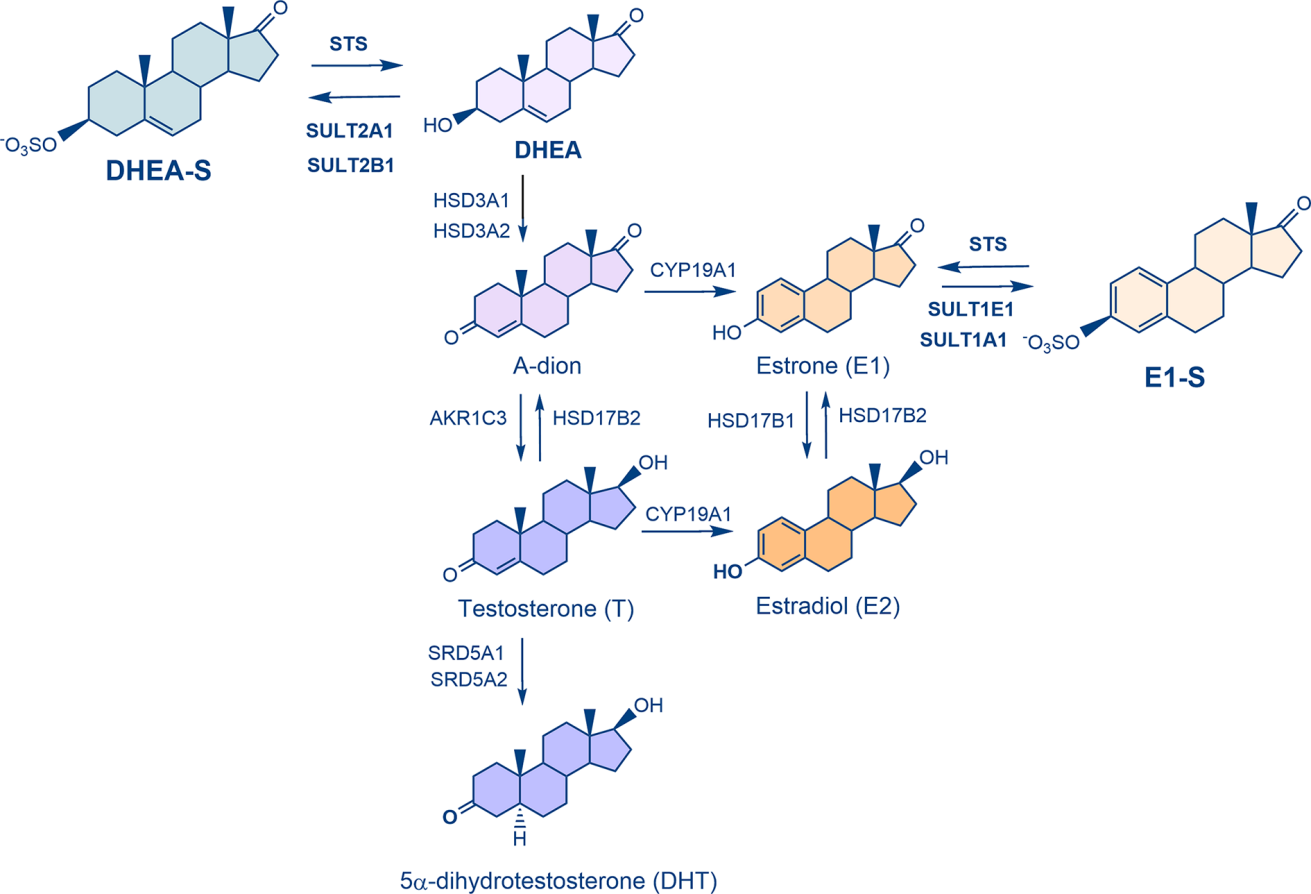
Biosynthesis of estrogens and androgens from inactive precursors DHEA-S and E1-S The most potent estrogen, estradiol (E2), can be formed from E1-S by the action of STS and 17β-hydroxysteroid dehydrogenases (HSD17B) (sulfatase pathway) or from DHEA-S, DHEA or androstenedione by the action of STS, 3β-hydroxysteroid dehydrogenases-Δ^5-4^ isomerases (HSD3B1 or HSD3B2), aromatase (CYP19A1) and HSD17B enzymes (aromatase pathway). The most potent androgen, 5α-dihydrotesterone (DHT), can be formed from DHEA-S by the actions of STS, HSD3B1 or HSD3B2, aldo-keto reductase 1C3 (AKR1C3) and 5α-reductases types 1 or 2 (SRD5A1 and SRD5A2).

In peripheral tissues, active estrogens and androgens can be formed from inactive or less active precursor steroids, mainly estrone sulfate (E1-S), dehydroepiandrosterone sulfate (DHEA-S), DHEA and androstenedione (Figure 1). These precursor steroids, E1-S and DHEA-S, are present in relatively high blood concentrations, 0.6 nM and 1.6 μM, respectively, in postmenopausal women, a population with a high incidence of hormone-dependent cancers [6,12]. The local formation of estrogens and androgens depends on the uptake of the most abundant steroid precursors E1-S and DHEA-S by transporters from the organic anion-transporting polypeptide (OATP) and organic anion transporter (OAT) families [13]. On the other hand, their active transport out of the cell is carried out by ATP-binding cassette pumps (*ABC*) [14].

### Steroid sulfatase

**Figure 2:**
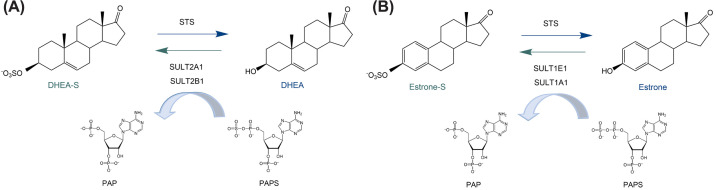
STS and SULT interconvert E1-S and E1, DHEA-S and DHEA Steroid sulfatase catalyzes the hydrolysis of DHEA-S (**A**) and E1-S (**B**), while sulfotransferases (SULT) catalyse the reverse reaction and require 3-phosphoadenosine-5-phosphosulfate (PAPS) as a sulfate donor.

Steroid sulfatase (STS, E.C. 3.1.6.2) catalyzes the hydrolysis of E1-S and DHEA-S (Figure 2), cholesterol sulfate and pregnenolone sulfate to their active counterparts with the highest catalytic activity (*v*_max_/*K*_M_) for E1-S, followed by pregnenolone sulfate DHEA-S and cholesterol sulfate [15]. STS belongs to a protein family of formylglycine (FGly)-dependent sulfatases that includes 17 human sulfatases containing FGly, which is generated posttranslationally by the action of the FGly-generating enzyme (FGE) encoded by the sulfatase modifying factor 1 (*SUMF1*) gene [16]. Enzymes from this family act on various substrates, including glycosaminoglycans (dermatan sulfate, heparan sulfate and keratan sulfate), sulfolipids, but also unidentified substrates [17]. Only STS, also known as arylsulfatase C (ARSC), acts on steroidal substrates [16]. STS is ubiquitously expressed (https://www.proteinatlas.org), not only in female and male reproductive tissues, but also in the gastrointestinal tract, kidney, bladder, respiratory system, liver and gallbladder, muscle tissue, skin, brain, endocrine tissue, etc [18]. The expression and role of STS in human homeostasis and in the pathophysiology of hormone-dependent diseases have been extensively studied [6,19,20]. STS also has a role in metabolic diseases [21] and has recently been linked to neurodegenerative Parkinson’s and Alzheimer’s diseases [22].

The structure of STS and its catalytic mechanism of action have been determined [23,24]. The first crystal structure (PDB 1P49) was solved more than 20 years ago and revealed a globular catalytic domain and a transmembrane domain consisting of two α-helices that serve as a hydrophobic stem [25]. Recently, the high-resolution (2.0 Å) crystal structure was solved (PDB 8EG3) (22). Based on this structure the author suggest that STS acts as a trimer and is anchored to a leaflet of the endoplasmic reticulum membrane, but does not span the bilayer [24,26]. The structure identified N-acetylglucosamines bound to Asn47 and Asn333, a potential phosphate binding site at His142 and, importantly, an additional binding site at the intermolecular interface near the catalytic center that can accommodate reaction products, but not E1-S and DHEA-S. It was hypothesized that this site is an alosteric site that induces a conformational change upon binding of E1 or DHEA and affects STS activity. In addition, Ghosh suggested that phophate bound to His142 may represent the second alosteric site that could influence the binding of E1 or DHEA and the catalytic activity of the STS trimer [26].

STS is up-regulated in inflammation by the nuclear factor kappa-light-chain-enhancer of activated B cells (NFκB) [21]. A number of *STS* polymorphisms have been reported [6], but have not yet been associated with cancer. Published data indicate that the hydrolysis and activation of E1-S and DHEA-S is not solely dependent on *STS* expression but can be influenced by SNPs and requires the expression of *SUMF1* gene and FGE activity [27]. When STS is expressed and active, it may also be influenced by high levels of E1 and DHEA, as well as phosphate, that may act as allosteric regulators [26].

## Sulfotransferases

Sulfotransferases (SULT) catalyze the transfer of a sulfate group from a coenzyme 3-phosphoadenosine-5-phosphosulfate (PAPS) to a number of compounds. Cytosolic SULT act on hydroxyl or amine residues of endogenous and exogenous molecules, including hormones, neurotransmitters, bile acids and xenobiotics [28]. In humans, there are 13 SULT and 5 of them, SULT1A1, SULT1E1, SULT2A1, SULT2B1a and SULT2B1b, mainly catalyze the sulfation of steroids, including E1, E2, catecholestrogens, DHEA (Figure 2), androsterone, pregnenolone and cholesterol [21,27]. *SULT* genes are expressed to varying degrees in the liver, gastrointestinal tract, lung, kidney, adrenal gland and also in reproductive tract tissue (https://www.proteinatlas.org). SULT1E1 has the highest catalytic efficiency in sulfating estrone, estradiol and catechol estrogens, while SULT1A1 can sulfate estrogens with lower efficiency and SULT2A1, SULT2B1a and SULT2B1b act on DHEA [6]. It is important to note that SULT1E1 acts also on a number of drugs or drug metabolites, including ethinylestradiol, fulvestrant, toremifene and tamoxifen metabolites, tibolone metabolites and also the antidiabetic drug troglitazone [29].

In recent decades, a number of crystal structures have been solved for all five SULT enzymes, showing that these enzymes are dimers in which the binding of a substrate to one monomer can have alosteric effects on the second monomer [28]. Several missense polymorphisms have already been linked to cancer, namely polymorphisms in *SULT1A1* that increase the risk of oral cancer and EC, polymorphisms in *SULT1E1* that increase the risk of breast cancer, and polymorphisms in *SULT2B1* that are associated with esophageal cancer [30]. Recently 220 missense SNPs were identified in *SULT1E1*, and five of them near the substrate and PAPS binding sites were found to have significantly reduced catalytic activity in mutants [31]. A number of polymorphisms have also been reported for *SULT2A1* and *SULT2B1*, and the effects of the non-synonymous coding SNPs on the activity of the recombinant isoforms have been investigated [32].

The regulation of SULT enzymes has already been described [6]. SULT1E1 is induced by oxidative stress via nuclear factor erythroid 2-related factor 2 (Nrf2), a regulator of cellular stress resistance, as well as via other nuclear receptors [27,29,33]. Furthermore, the activity of SULT1E1 can be inactivated under oxidative stress by S-glutathionylation of Cys83 in the active site [27]. These data indicate that the sulfation of estrogens and androgens is not only influenced by altered *SULT* expression but can also be affected by SNPs, PAPS levels, covalent modifications and potential allosteric regulators.

## Sulfatases and sulfotransferases in endometrial cancer

EC is the most common gynecological cancer in the developed world with 417,367 new cases and 97,370 deaths reported in 2020 [1]. Traditionally, EC has been divided into well-differentiated type 1 and poorly differentiated type 2 with poorer prognosis. Four prognostically relevant molecular subtypes have been identified in the last ten years: polymerase epsilon mutated, mismatch repair defficient/MSI hypermutated, *TP53* wild type/low copy number, *TP53* mutated/high copy number [34].

Patients with EC have significantly higher circulating levels of E1, E2 and E1-S [35]. Several studies report that *STS* and *SULT1E1* are aberrantly expressed in EC at mRNA and protein levels [6,36,37]. However, both higher and unchanged STS levels have been found in EC compared with control endometrium [6,36,37]. We observed increased E1-S metabolism to E1 and E2 in EC compared with adjacent control tissue, no difference in STS mRNA and protein levels, and large interindividual variability with decreased immunoreactivity based on immunohistochemical staining in EC using specific and validated antibodies (*n*=44) [38]. It has been reported that *SULT1E1* expression was unchanged or up-regulated in EC compared with control endometrium at mRNA and protein levels [6,36,37]. We found significantly lower SULT1E1 mRNA levels in EC compared with adjacent control endometrium only in premenopausal patients, almost no expression at the protein level and again large interindividual variability in immunoreactivity (*n*=44) [38]. Cornel et al. found no difference in mRNA and protein levels of STS and SULT1E1, but lower STS activity in EC tissue (*n*=31) compared with control endometrium of postmenopausal patients with benign disease (*n*=19) [38]. Furthermore, in their study, the ratio of STS/SULT1E1 activities was significantly lower in EC tissue compared with control endometrium [39]. Discrepancies in the data published to date can be explained by differences in menopausal status, control tissue (adjacent control tissue from the same patients versus control tissue from patients with benign pathology), variability among EC patients (effects of cancer differentiation and grade, molecular subtypes, tumor heterogeneity), but also by the use of suboptimal antibodies [40]. Currently, the importance of estrogen production *via* the sulfatase pathway is supported by decreased levels of E2, E1 and E1-S in postmenopausal patients after surgery, an increased risk of recurrence in patients with higher E1-S levels (41) and also by the conversion of E1-S to E1 and E2 in EC tissue [38].

**Figure 3:**
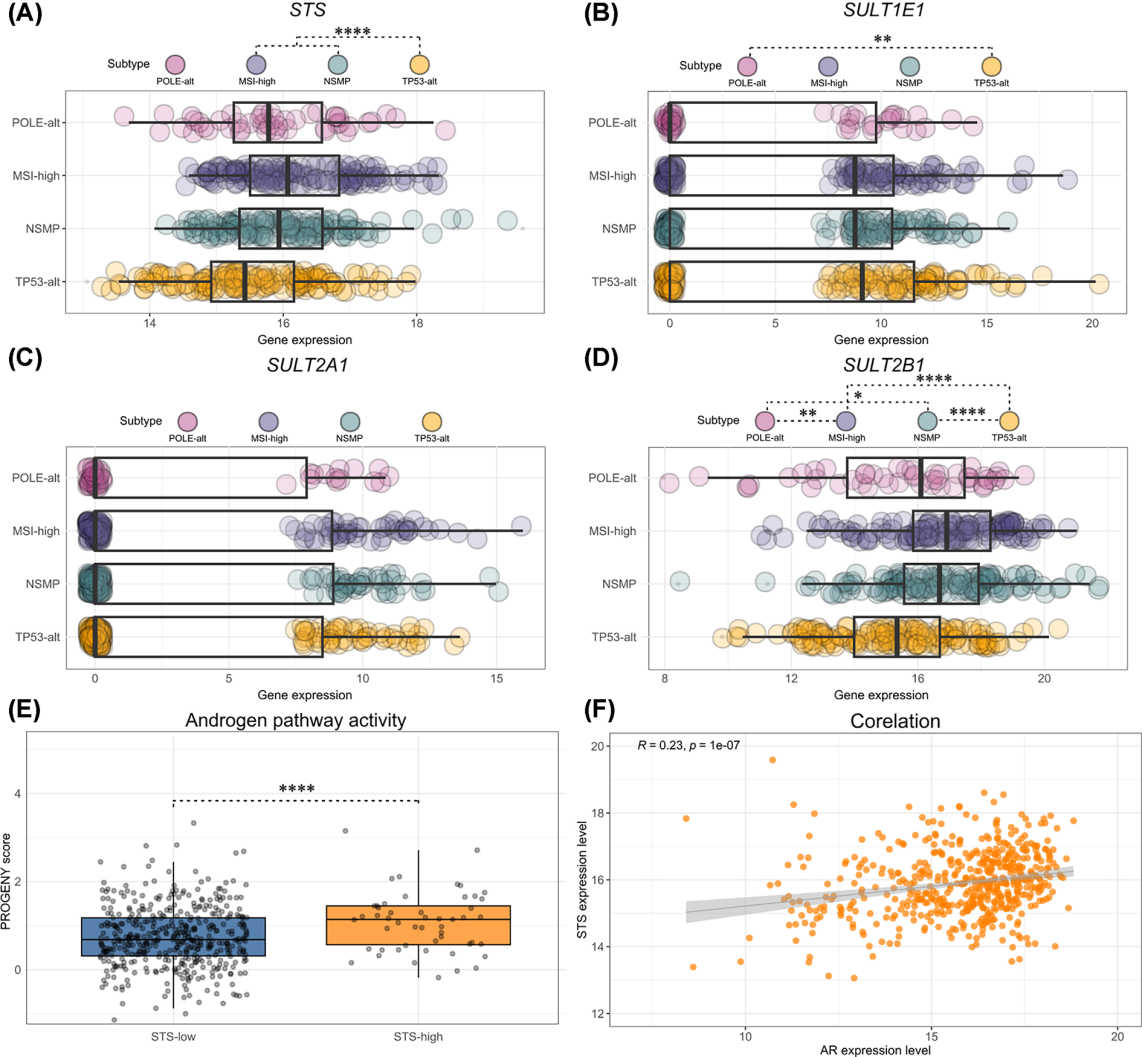
STS, SULT and AR in EC – analysis of TCGA data Analysis of TCGA EC transcriptome data from patients stratified into molecular subtypes; POLE-altered (*n*=48), MSI-high (*n*=142), NSMP (*n*=141) and TP53-altered (n = 151). Boxplots illustrate the expression of *STS* (**A**), *SULT1E1* (**B**), *SULT2A1* (**C**) and *SULT2B1* (**D**). Androgen pathway activity in endometrial tumors with low *STS* (*n*=428) and high *STS* (*n*=54) expression, estimated with the Progeny package in R Studio (**E**). Correlation between *STS* and *AR* expression in EC (*n*=482) (**F**). Gene expression is expressed as log2(FPKM-uq+1). Data are presented as boxplots showing the median, first and third quartiles and whiskers as min-max values and the raw data as individual points. Significance levels: *P<0.05, **P<0.01,****P<0.0001 by Kruskal−Wallis followed by Dunn’s post-hoc test with Bonferroni correction (A−D), Mann−Whitney *U* test (E), Spearman’s rank correlation coefficient ρ (**F**). FPKM, fragments per kilobase of transcript per million mapped fragments; MSI, microsatellite instability; NSMP, non-specific molecular profile; POLE, polymerase ε; uq, upper quartile.

STS and SULT are also important for androgen biosynthesis [4]. EC patients with longer survival have higher serum levels of DHEA-S and DHEA [41], which could influence local androgen formation. Moreover, *AR* expression correlates with longer survival, and a recent meta-analysis confirmed its prognostic value [42], so androgens may play a protective role. We have shown that genes encoding the enzymes required for the metabolism of DHEA-S and DHEA to active androgens are expressed in EC, albeit without *SULT2A1* and with higher levels of *SULT2B1* [6] and with up-regulation of several DHEA-S uptake transporters from the OATP family [43,44]. AKR1C3, a key enzyme of androgen metabolism, is also expressed and correlates with better survival of EC patients [45] and our unpublished studies have shown that DHEA-S is metabolized to active androgens in EC cell lines (Gjorgoska and Rizner). All these data support the importance of STS and SULT in the biosynthesis and action of androgens, with these enzymes acting in concert with uptake/efflux transporters to control intracellular androgen levels. To better understand the role of these enzymes in the pathophysiology of EC, we analysed TCGA transcriptomics data (*n*=482) and found a positive correlation between *STS* and *AR* expression and higher androgen pathway activity in patients with high *STS* expression, with the lowest *STS* and highest *SULT2B1* expression in the most severe EC (*TP53* mutated) (Figure 3). These data suggest that STS plays a role in androgen formation only in non-*TP53*-mutated cancers.

## Sulfatases and sulfotransferases in ovarian cancer

OC is the deadliest of the hormone-dependent cancers. Worldwide, 313,959 new cases and 207,252 deaths were reported for this gynecologic cancer in 2020 [1]. It is estimated that the incidence of OC will increase by 55% and the number of deaths by 67% by 2035 (World Ovarian Cancer Coalition 2018). OC is traditionally divided into epithelial and non-epithelial cancers, with serous epithelial cancers (SOC) accounting for 70% of all cancers. The most common and aggressive cancer is high-grade serous ovarian cancer (HGSOC) [46]. It is generally recognized that the different histotypes of OC have different origins and only have the ovary as an anatomical site in common. Low-grade ovarian cancer originates from the surface epithelium of the ovary and HGSOC from the fallopian tubes [47]. HGSOC is also categorized into four molecular subtypes, including immunoreactive, differentiated, proliferative and mesenchymal with different overall survival (OS) [48].

More and more data indicate that OC is estrogen-dependent. The WHI and Million Women epidemiologic studies [48,49] suggest that both estrogen-only and estrogen-progestin hormone replacement therapies increase OC risk [50], and other studies support associations between estrogens and estrogen-DNA adducts and various OC histotypes [51,52]. Androgens may also influence ovarian carcinogenesis via activation of the androgen receptor (AR) or through their role as estrogen precursors. AR is expressed in 40% of benign epithelial neoplasms and 64% of OC [53]. Androgens thus appear to play a direct role in pathophysiology but have not yet been adequately studied. Our unpublished data show that DHEA-S is metabolized to androgens in HGSOC cell lines (Gjorgoska and Rizner).

**Figure 4:**
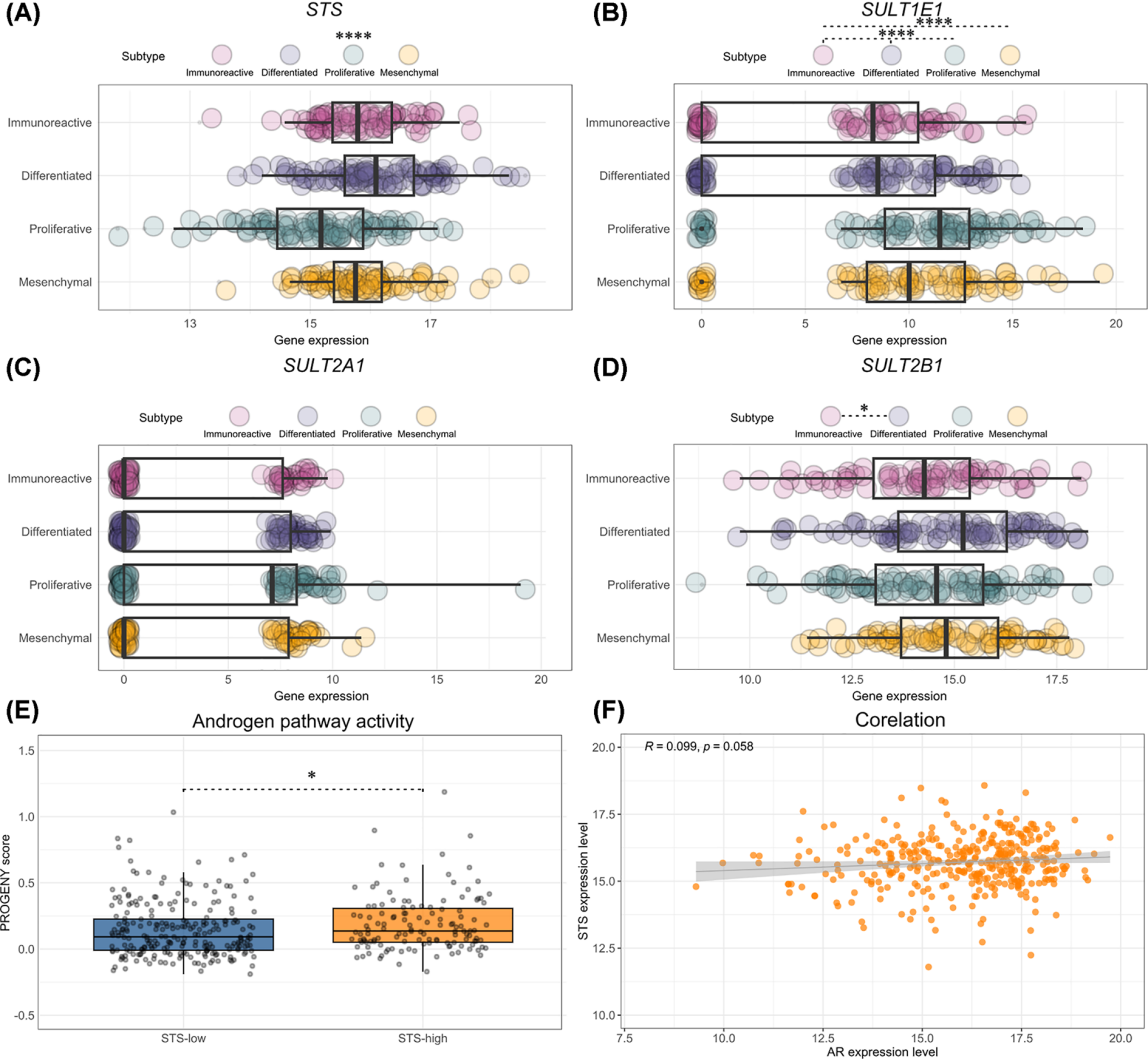
STS, SULT and AR in HGSOC – analysis of TCGA data Analysis of TCGA HGSOC transcriptome data from patient stratified into molecular subtypes; immunoreactive (*n*=80), differentiated (*n*=98), proliferative (*n*=99) and mesenchymal (*n*=87). Boxplots illustrate the expression of *STS* (**A**), *SULT1E1* (**B**), *SULT2A1* (**C**) and *SULT2B1* (**D**). Androgen pathway activity in HGSOC with low-*STS* (*n*=128) and high-*STS* (*n*=236) expression, as estimated using Progeny package in R Studio (**E**). Correlation between *STS* and *AR* expression in HGSOC (n = 364) (**F**). Gene expression is expressed in log2(FPKM-uq+1). Data are represented as boxplots showing the median, first and third quartiles and whiskers as min-max values and the raw data as individual points. Significance levels: *p<0.05, ****p<0.0001 by Kruskal-Wallis followed by Dunn’s post-hoc test with Bonferroni correction (A−D), Mann−Whitney *U* test (E), Spearman’s rank correlation coefficient ρ (**F**). FPKM, fragments per kilobase of transcript per million fragments mapped; HGSOC, high-grade serous ovarian cancer; uq, upper quartile.

There are few data on the expression and activity of STS and SULT1E1 in OC tissue samples. STS has been detected to varying degrees in different histotypes of OC [6]. The largest study to date investigated STS and SULT1E1 immunoreactivity in 206 patients with serous and non-serous histology. In 137 HGSOC patients, SULT1E1 acted as an independent prognostic factor for OS, with a hazard ratio (HR) of 0.66, while STS and ERα had no significant impact on survival [54]. Our recent analysis of publicly available HGSOC transcriptomics (*n*=300) and proteomics data (*n*=252) showed weak *SULT1E1* expression [55]. *ESR1* was expressed at significantly higher levels than *ESR2* and *GPER* [55], suggesting that active estrogens would act via ERα [55]. At the protein level, we found higher STS levels compared with SULT levels. Interestingly, four molecular HGSOC subtypes differed in the expression of SULT1E1 with the highest protein levels found in proliferative subtype [55]. For STS, recent studies showed no association with different OC histotypes (*n*=147) and stage of disease, but lower OS in patients whose tumor tissue stained positive for both STS and AR [56]. Our further analysis of TCGA transcriptomics data revealed higher androgen pathway activity in HGSOC patients (*n*=364) with high *STS* expression, poor correlation between *STS* and *AR* expression and weak *SULT2A1* expression with a large inter-individual variability. The proliferative subtype had the lowest *STS* expression and the highest *SULT1E1* and *SULT2A1* expression (Figure 4). This finding supports the importance of intratumoral androgen biosynthesis and action in non-proliferative HGSOC subtypes.

## Sulfatase and sulfotransferase inhibitors and clinical trials

STS has been recognized as a target for the treatment of hormone-dependent diseases, particularly breast and prostate cancer, which has led to the synthesis of a number of steroidal and non-steroidal inhibitors that have been the subject of several reports [57,58]. To date, STS inhibitors have been tested in clinical trials in patients with breast cancer, EC, endometriosis and prostate cancer, while no clinical trial has been conducted in OC [7]. Recently, STS has also been associated with neurological diseases. Lower DHEA-S levels have been measured in patients with Parkinson’s disease and dementia [21], thus STS inhibitors may also be beneficial in these conditions. Interestingly, increased levels of sulfated steroids elevated longevity in *Caenorhabditis elegans* and decreased proteotoxicity in *C. elegans* models of neurodegenerative diseases [59].

In a phase II clinical trial in ER-positive advanced/refractory EC patients, the irreversible non-steroidal STS inhibitor STX64 performed worse than the progestin megestrol acetate [6,38]. This finding and more recent studies suggesting that androgens may play a protective role in EC call into question the suitability of STS inhibitors for the treatment of EC. Unfortunately, data on *STS* expression and molecular subtypes of the included patients are not available for this clinical trial. Based on the analysis of the TCGA data, the *TP53* mutated EC subtype would be expected to have the least impact on intratumoural estrogen and androgen biosynthesis. Accordingly, STS inhibitors in HGSOC patients might only have effects on estrogen and androgen formation in non-proliferative subtypes. Further studies are needed to assess whether these effects would benefit patients.

STS inhibition would increase the levels of E1-S and DHEA-S, which acts as a neurosteroid and thereby activates a number of ion channels [60], and also affects the phosphorylation of the kinase Erk1/2 and the transcription factors CREB and AFT-1, probably via an as yet unknown G protein-coupled receptor [61]. STS inhibition would reduce DHEA levels, which also has a variety of tissue-specific effects. In prostate and cervical cancer cell lines DHEA stimulates epithelial−mesenchymal transition, cell migration, invasion, and aerobic glycolysis. DHEA also has beneficial effects by improving vascular endothelial function, restoring insulin sensitivity and reducing plasma triglycerides and inflammatory cytokines, thus protecting against metabolic disorders [21,62,63].

On the other hand, SULT enzymes can be inhibited by various xenobiotics, including non-steroidal anti-inflammatory drugs [64] and SULT1E1 and SULT1A1 are also inhibited by the hydroxylated product of environmental pollutants, polychlorinated biphenyls [65] and phthalates [66], which may affect local estrogen and androgen levels and may influence the risk of carcinogenesis.

## Sulfatase and sulfotransferases as prognostic biomarkers for EC and OC?

There are only a few studies that have investigated the prognostic properties of STS and SULT or their substrates in EC and OC. The study of 126 postmenopausal EC patients showed that these patients had higher estrogen and androgen levels compared with healthy women, with the highest estrogen levels in low-grade and less invasive cancers and the highest E1-S serum levels in patients without myometrial invasion [35]. In contrast, patients with recurrence had 2-fold higher E1-S levels than patients without recurrence [35]. These findings were confirmed in a larger group of 246 EC patients in which higher preoperative E1-S levels were associated with a higher risk of recurrence (HR = 2.67, 95% CI = 1.02–6.99; *P*=0.045) [67], suggesting that E1-S and concurrent STS and SULT1E1 may serve as prognostic biomarkers. However, in a study of 59 pre- and postmenopausal EC patients, Lee at al. found no association between imunohistochemical STS levels and progression-free survival (PFS) or OS [68], and this was also confirmed by our analysis of TCGA data (Gjorgoska, Rizner, unpublished).

In OC, Chura et al. confirmed STS activity in 97% of samples from 37 epithelial OC patients treated with platinum-based therapy. They showed that in advanced-stage OC, increased STS activity is associated with poorer PFS. The median PFS was significantly shorter in patients with high STS activity than in patients with low activity (6.9 and 23.5 months, respectively, *P*=0.008) [69]. These findings were confirmed in a recent study of 154 epithelial pre and post-menopausal OC patients in which STS was associated with shorter OS of patients (log-rank test, *P*=0.032). The univariate and multivariate Cox proportial hazard regression analyzes revealed a significantly higher HR in AR-positive tumors with STS expression (HR = 3.46, *P*=0.049 and HR = 5.92, *P*=0.0199) compared with AR-negative tumors, suggesting that simultaneous expression of AR and STS predicts poor prognosis in epithelial ovarian cancers [56]. In the present study, however, patients were not stratified according to their histology. In 67 postmenopausal women with HGSOC, a univariate Cox analysis showed that ER pathway activity favored disease-free survival (DFS) (HR = 0.943, *P*=0.033) and OS (HR = 0.894, *P*=0.041), but not in premenopausal patients [70]. In another study including 132 advanced-stage HGSOC, multivariate Cox analysis identified SULT1E1 as an independent predictor of OS (HR = 0.66, *P*=0.005), while STS and ERα had no effect on survival [54]. Interestingly, higher STS immunoreactivity was recently associated with longer OS in breast cancer, with an inverse association between STS and ERα [71].

## Concluding remarks and perspectiveses

**Figure 5:**
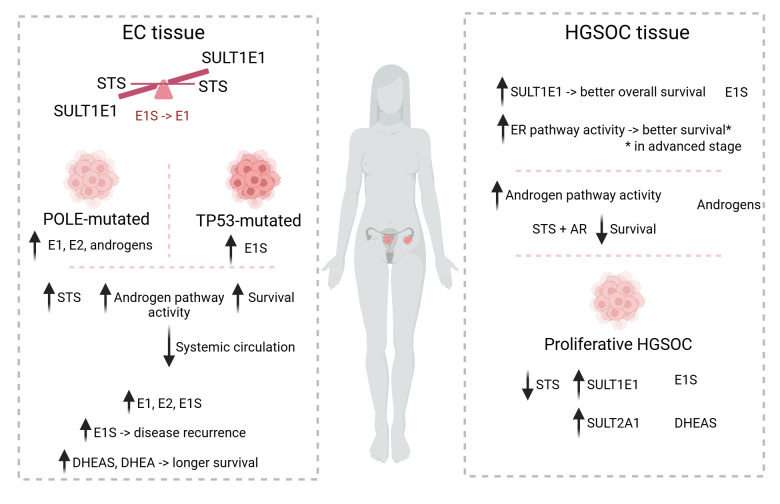
STS and SULT in EC and HGSOC Scheme summarising the current state of knowledge on the expression of *STS* and *SULT* in EC and HGSOC. Data are shown for POLE-mutated and TP53-mutated EC, with good and poor prognosis, respectively, and for HGSOC and the particularly proliferative subtype with relatively poor prognosis.

This review provides an overview of the current status of STS and SULT in EC and OC as well as new findings from the analysis of TCGA data (Figure 5). At the same time, it highlights a number of questions/issues that need to be addressed in future studies.

When assessing the importance of STS and SULT in pathopysiology, it is important to note that the activities of these enzymes depend on several factors: (i) presence of SNPs, (ii) covalent modifications (FGly in STS), (iii) presence of cofactors (PAPS in SULT), (iv) potential allosteric regulation (STS and SULT1E1) and (v) intacellular redox state (inactivation by S-glutathionylation in SULT1E1). Thus, the expression *per se* do not provide sufficient information. Measuring STS and SULT activities in tissue samples would better reflect the physiological/pathophysiological context. In the future, the impact of cancer differentiation and grade, molecular subtypes and tumor heterogeneity should be considered and STS and SULT need to be investigated in different histological and molecular subtypes of these cancers. In addition to STS and SULT, their SNPs, protein levels using validated antibodies and especially enzymatic activities, and the expression of FGE, should also be investigated. Furthermore, the DHEA-S and E1-S uptake/efflux transporters also play a decisive role and therefore need to be systematically studied. All in all, the role of STS and SULT in EC and OC remains to be further investigated, and given the potential benefits of STS inhibitors, the jury is still out.

## Summary

- STS and SULT regulate peripheral estrogen and androgen biosynthesis from the inactive precursors DHEA-S and E1-S and are thus implicated in hormone-dependent diseases.
- In EC and OC, the concentrations of active estrogens and androgens depend on DHEA-S and E1-S uptake transportes and the balance between STS and SULT enzymes.
- In EC, E1-S metabolism proceeds in well-differentiated cancers, and high levels of E1-S predict disease recurrence. There is a positive correlation between STS and AR expression, which is a known positive predictive factor.
- In HGSOC, SULT1E1 acts as an independent predictor of overall survival. Higher STS expression is associated with higher androgen pathway activity.
- To understand the exact role of STS and SULT enzymes in the pathophysiology of EC and OC, further studies are needed to evaluate their expression and activities in all molecular subtypes of these cancers.

## Abbreviations

11-keto T: 11-ketotestosterone
11-keto-DHT: 11-ketodihydrotestosterone
ABC: ATP-binding cassette transporter
AFT-1: iron-regulated transcriptional activator 1
AKR1C3: aldo keto reductase 1C3
ARSC: arylsulfatase C
CREB: cAMP response element-binding protein
CYP11B1: 11β-hydroxylase
CYP19A1: aromatase
DFS: disease free survival
DHEA: dehydroepiandrosterone
DHEA-S: dehydroepiandrosterone-sulfate
DHT: 5α-dihydrotestosterone
E1: estrone
E1-S: estrone sulfate
E2: estradiol
EC: endometrial cancer
ER: estrogen receptor
ERK1/2: extracellular signal regulated kinase
ESR1: gene encoding ERα
ESR2: gene encoding ERβ
FGE: formylglycine-generating enzyme
FGly: formylglycine
FPKM: fragments per kilobase of transcript per million mapped fragments
GPCR: G protein-coupled receptor
GPER: G protein-coupled estrogen receptor
HGSOC: high grade serous ovarian cancer
HR: hazard ratio
HSD11B: 11β-hydroxysteroid dehydrogenase
HSD17B: 17β-hydroxysteroid dehydrogenase
HSD3B: 3β-hydroxysteroid dehydrogenase-Δ^5-4^ isomerase
LGSOC: low grade serous ovarian cancer
MSI: microsatellite instability
NFκB: nuclear factor kappa-light-chain-enhancer of activated B cells
Nrf2: nuclear factor erythroid 2-related factor 2
NSMP: non-specific molecular profile
OAT: organic anion transporter
OATP: organic anion transporting polypeptide
OC: ovarian cancer
OS: overall survival
PAP: 3-phosphoadenosine-5-phosphate
PAPS: 3-phosphoadenosine-5-phosphosulfate
POLE: polymerase ε
SNP: single nucleotide polymorphism
SRD5A: 5α-reductase
STS: sulfatase
SULT: sulfotransferase
SUMF1: sulfatase modifying factor 1
TP53: tumor protein P53
